# Exploring Vulval Fibroadenoma: A Rare Entity

**DOI:** 10.7759/cureus.78714

**Published:** 2025-02-07

**Authors:** Anu Berwal, Komali Kancharla

**Affiliations:** 1 Obstetrics and Gynaecology, Maharaja Agrasen Medical College, Agroha, IND

**Keywords:** case report, controversial origin, ectopic breast tissue, fibroadenoma, mammary-like anogenital glands, vulval mass

## Abstract

Vulval fibroadenoma is a rare benign neoplasm mimicking mammary fibroadenoma, with limited cases reported earlier. Its origin is still debatable, linked to either ectopic mammary tissue or specialized anogenital glands. Similar to breast fibroadenoma, it usually presents as a painless, firm, well-circumscribed mass and responds to hormonal changes during menstruation, pregnancy, or lactation. Its diagnosis relies on core needle biopsy or fine needle aspiration cytology, while treatment involves surgical excision with clear margins to prevent a recurrence. We report a 33-year-old woman with a painless pedunculated vulval mass hanging from the right labia majora. Ultrasonography showed a heterogeneous lesion with mild internal vascularity. Fine needle aspiration cytology was advised, but the patient opted for immediate removal. Excised mass on microscopy revealed glandular and stromal hyperplasia with cuboidal cells resting on a myoepithelial layer, and stroma contained spindle cells arranged in fascicles with elongated nuclei and moderate cytoplasm along with peri-canalicular pattern, suggestive of a fibroadenoma. No recurrence was noted during a one-year follow-up. This case of vulval fibroadenoma emphasizes the importance of considering ectopic breast tissue or mammary-like anogenital glands in the differential diagnosis of vulval masses. Although there are more case reports, it still remains uncommon with varied presentations, necessitating further discussion.

## Introduction

Vulval fibroadenoma is a rare benign neoplasm, with only a limited number of cases documented in the literature so far. It is a fibroepithelial lesion that mimics breast fibroadenoma. Its histogenesis remains a subject of ongoing debate, with two primary theories proposed: the milk line theory and the mammary-like anogenital glands (MLAG) theory. These theories suggest that this vulval mass may arise from remnants of ectopic mammary tissue or specialized glandular structures found in the anogenital region [1-5]. Ectopic breast tissue is a rare phenomenon, with the axilla being the most common and the vulva being the second most common site [6]. The condition is most commonly seen in women aged 20 to 80 years [7], often presenting after puberty, and tends to enlarge in response to hormonal changes, such as those occurring during menstruation, pregnancy, and lactation, due to the presence of hormone receptors [1-4]. Clinically, vulval fibroadenomas are similar to breast fibroadenomas, both in presentation and histopathological features. Diagnosis is typically made through fine needle aspiration cytology or core needle biopsy, which is a valuable tool for preoperative evaluation. Treatment involves surgical excision with clear margins to minimize the risk of recurrence. In cases where malignancy is suspected, immunohistochemical studies for hormone receptors may provide further diagnostic and prognostic insight. We present a case of a 33-year-old woman who presented with a pedunculated vulval mass. She underwent surgical excision, and a histopathological examination revealed the diagnosis of vulval fibroadenoma. This case underscores the importance of considering vulval fibroadenoma in the differential diagnosis of vulval masses. It highlights the role of surgical intervention and histopathological analysis in managing this rare condition.

## Case presentation

**Figure 1:**
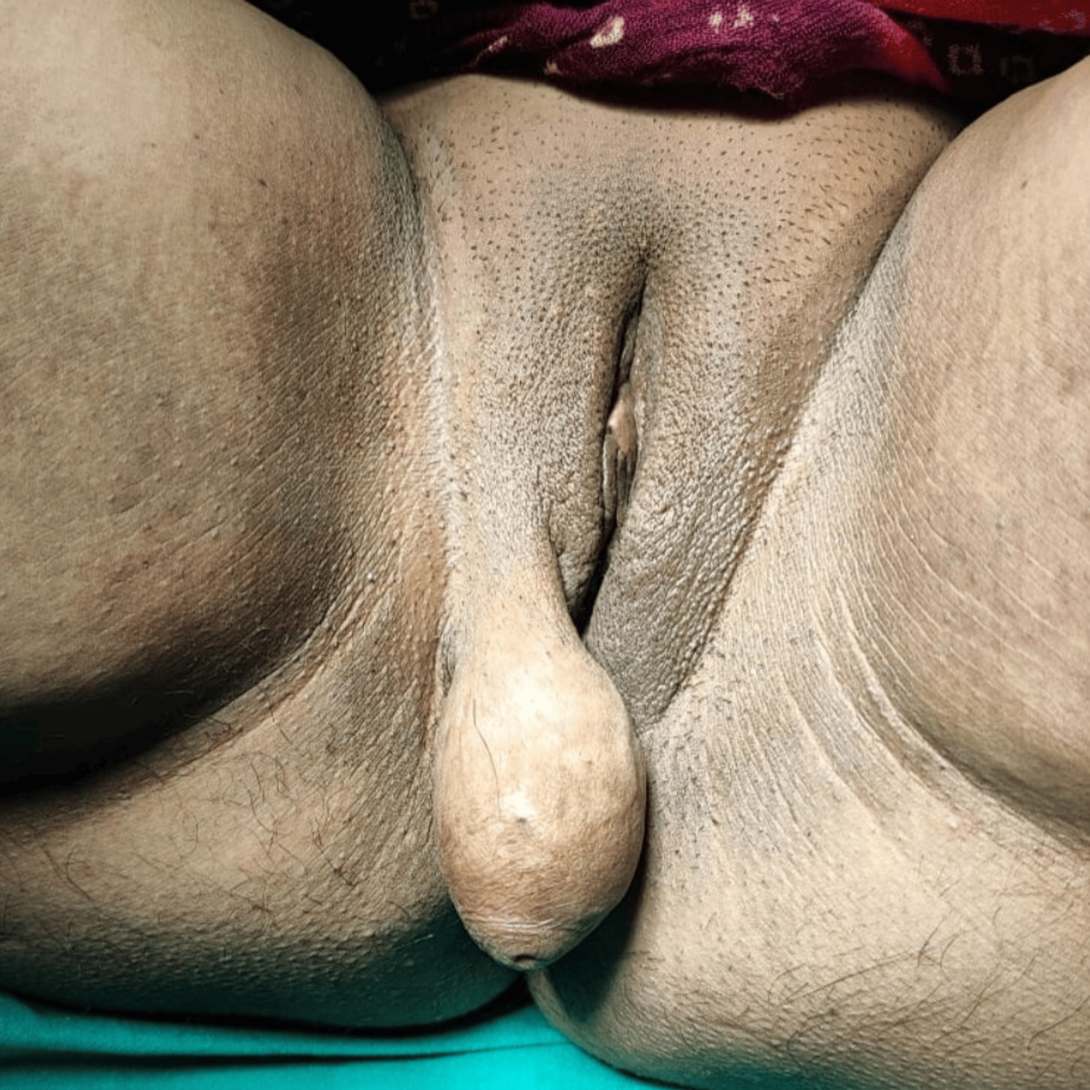
Pedunculated vulval mass hanging from right labium majus

A 33-year-old P2L2 woman presented at the gynecology outpatient department with a painless vulval mass present for four years. Initially, the size of a grape, the mass enlarged over time to the size of a lemon. There was no history of any hormonal drug intake or menstrual irregularities. No history of tobacco or alcohol consumption or smoking was there. The patient had no significant family history. On physical examination, a pedunculated mass measuring approximately 5×4×3 cm was observed, hanging from the right labia majora with a 2-3 cm long stalk. The mass was ovoid, smooth, firm, not compressible, and nontender. It was not fixed to underlying structures, the cough impulse was negative, and no signs of ulceration, color changes, puncta, or secretions were seen (Figure 1). Breast, abdominal, inguinal, and gynecologic examinations were unremarkable. The pap smear was normal. Abdominopelvic sonography was normal, while ultrasonography of the mass revealed a heterogeneous lesion with mild internal vascularity. A probable diagnosis of vulval leiomyoma was made.

**Figure 2:**
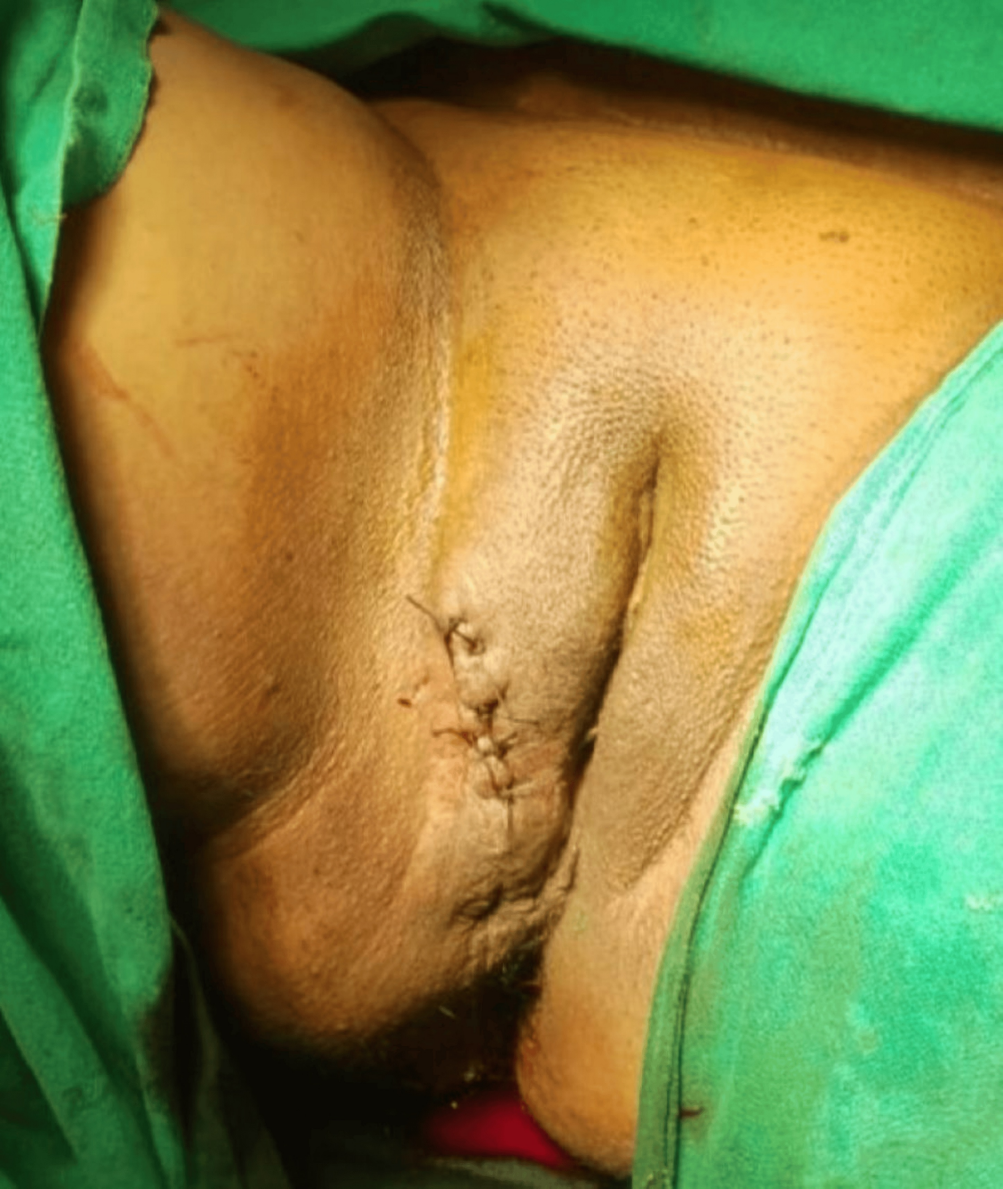
Picture after excision of right sided vulval mass

Prior fine needle aspiration cytology was advised, but the patient opted directly for complete removal. Considering the probably benign outlook of the mass, excision of the mass was performed under local anesthesia. Clamps were applied at the base of the peduncle, and mass was excised (Figure 2).

**Figure 3:**
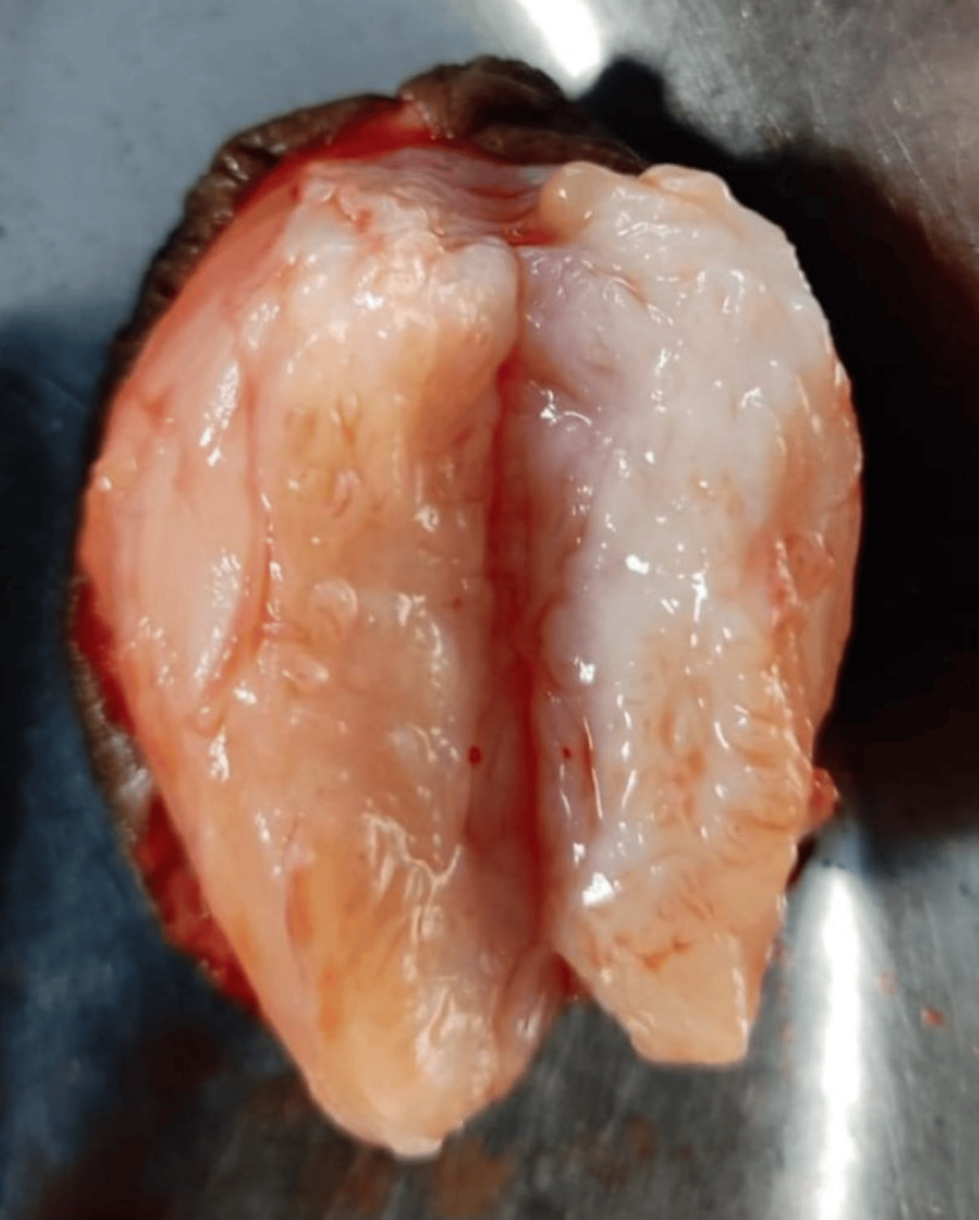
Gross photograph showing cut section of oval pink tanned soft tissue with slit like areas covered externally by skin.

**Figure 4:**
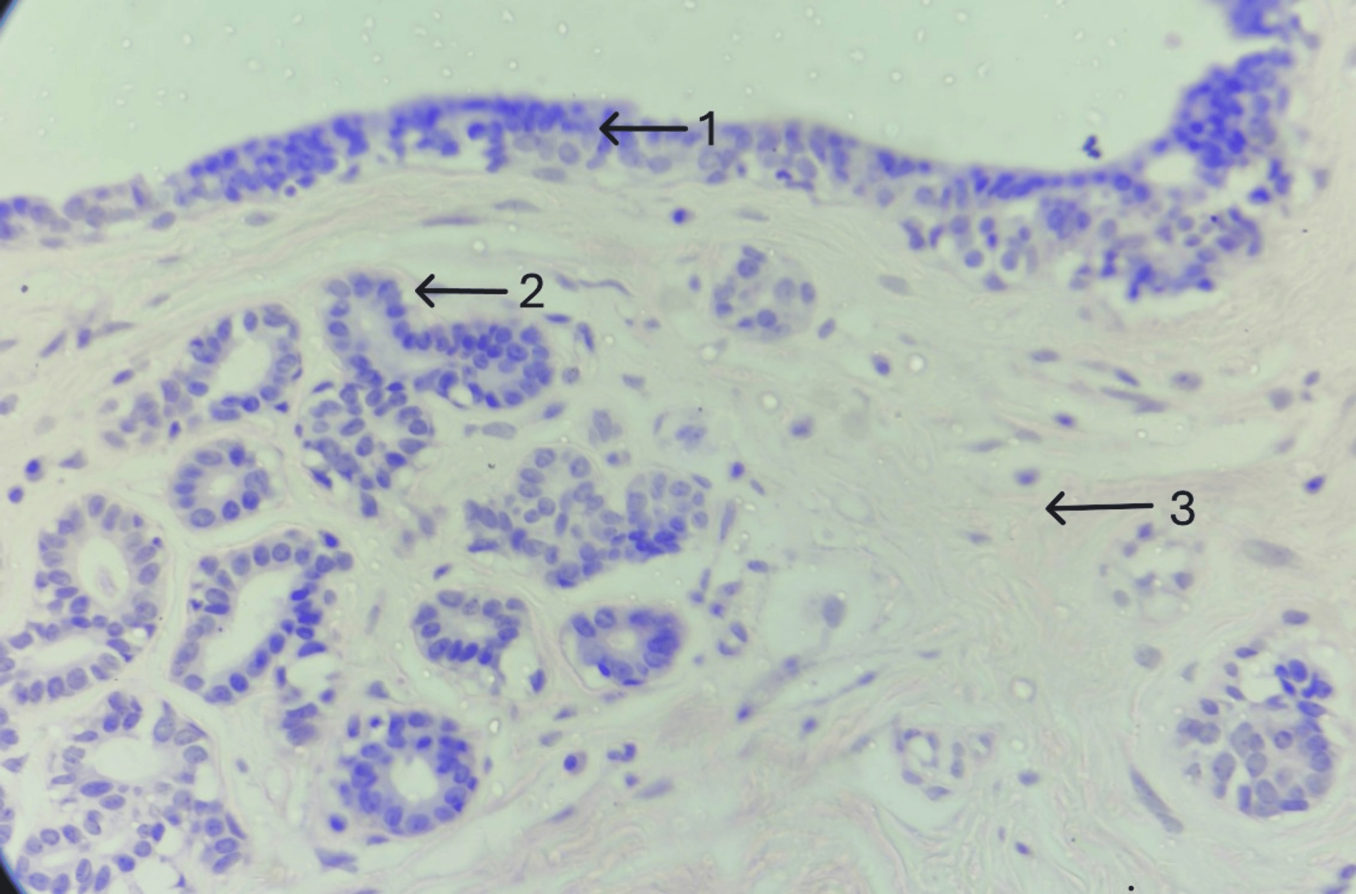
A well circumscribed biphasic tumor. H&E 40x: hematoxylin and eosin; arrow 1 shows stratified squamous epithelium; arrow 2 shows glandular hyperplasia; arrow 3 shows stromal hyperplasia.

**Figure 5:**
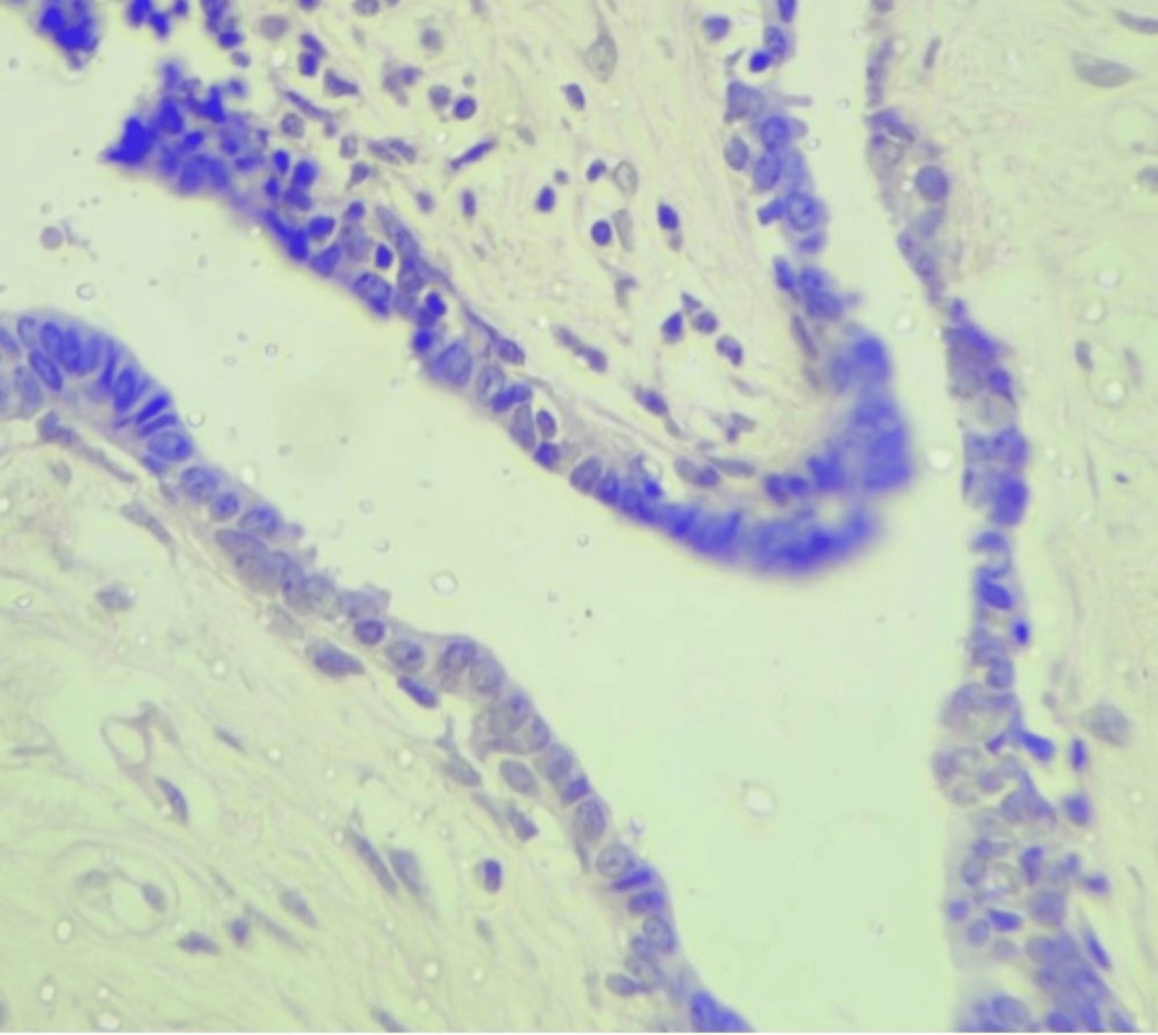
A bilayered epithelium comprising of inner cuboidal to columnar epithelium and outer myoepithelium. H&E 40x: hematoxylin and eosin.

**Figure 6:**
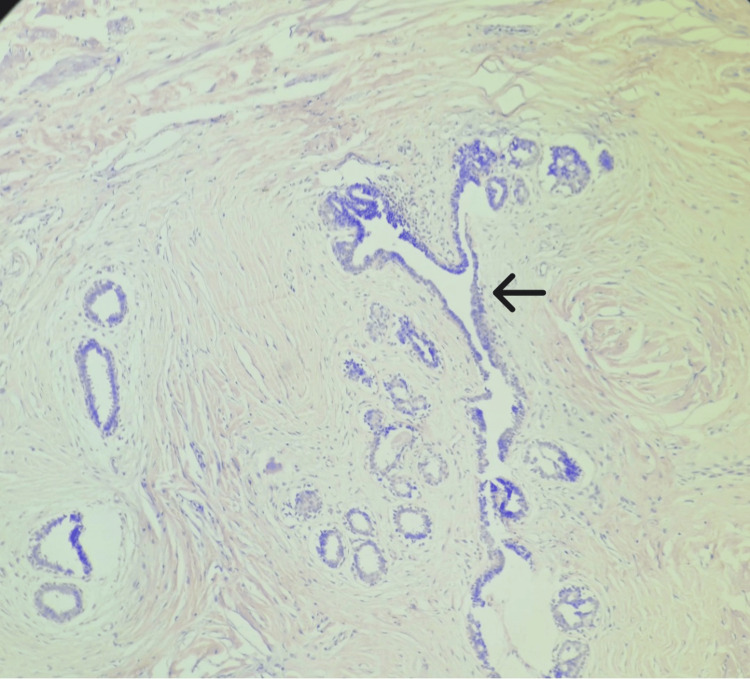
Well circumscribed neoplasm with intact lobular architecture. H&E 10x: hematoxylin and eosin; arrow depicts pericanalicular growth pattern.

Gross examination revealed a 5.5×4.5×3.5 cm firm globular mass covered by skin. On the cut section, a homogenous, pink-tanned soft tissue mass with slit-like areas was observed, with no cystic changes, hemorrhage, or necrosis (Figure 3). Microscopic analysis showed glandular and stromal hyperplasia (Figure 4). Glands were composed of cuboidal cells with round, regular nuclei supported by a myoepithelial cell layer (Figure 5). The stroma consisted of spindle cells arranged in fascicles with elongated nuclei and moderate cytoplasm. A pericanalicular pattern was also noted (Figure 6). No evidence of atypia was seen. Based on these findings, a histological diagnosis of vulval fibroadenoma was established.

The patient was followed up for one year, showing no recurrence or complications.

## Discussion

Vulval fibroadenomas are rare benign tumors that present unique challenges in diagnosis and management. Their epidemiology remains largely underexplored due to the limited number of reported cases. Typically, vulval fibroadenomas are associated with either ectopic mammary tissue (EBT) along the embryological milk line or mammary-like anogenital glands (MLAG). The first documented case of ectopic mammary tissue was reported by Hartung in 1872, as discussed by Garcia et al. [8]. Kajawa classified accessory breast tissue into eight classes as per various forms, ranging from complete breasts with nipples to the presence of only nipples [9]. EBT theory suggests that these tissues are predominantly located along the milk line, but rare presentations have been observed in regions such as the vulva, shoulders, buttocks, and ears. The most common site, however, is the axilla, accounting for 60%-70% of the cases [10]. The overall incidence of EBT is around 2%-6% in females and 1%-3% in males, with variations among different races, most commonly seen in Japanese women. Primary breast cancer in EBT is reported in 0.3%-0.6% of all breast cancers [11]. The most common problems seen with ectopic breast tissue include fibrocystic changes or mastitis.

The alternative theory, proposed by Putte in 1994, suggests the presence of mammary-like glands in the anogenital region. He discussed that the extension of the mammary ridge to the vulva has not been proven definitely, and the milk line theory cannot explain perineal ectopic breast tumors [5]. The anogenital glands he described are closely related to eccrine glands and share histological similarities with mammary tissue. This concept is supported by our case, where the lesion was histologically consistent with fibroadenomas arising from mammary-like anogenital glands, as no mammary tissue was seen. Carter et al. have highlighted limitations in confirming histogenesis due to insufficient documentation of surrounding tissue in most of the studies, as the well-defined nature of the lesion allows for simple excision. A more extensive resection may be needed for thorough histological analysis [12].

The present case of vulval fibroadenoma offers several unique clinical features compared to previously documented cases. Our patient’s fibroadenoma displayed a distinct pedunculated structure, unlike the more commonly reported nodular or subcutaneous lesions. While some cases describe pain or discomfort, particularly when associated with hormonal stimulation or malignancy, this mass was painless. Usually, vulval fibroadenomas are linked to hormonal fluctuations during pregnancy, lactation, or other hormonal states, but our case presented without any typical physiological or hormonal associations.

Given the rarity of vulval fibroadenomas, differential diagnoses of a vulval mass should include a variety of conditions, ranging from benign lesions like epidermal cysts, Bartholin’s cysts, and lipomas to more complex conditions such as phyllodes tumors, fibrocystic disease, apocrine adenomas, hidradenoma papilliferum, extra-mammary Paget’s disease, and mucinous adenocarcinoma. A vulval leiomyoma was kept as a probable diagnosis in our case, considering the well-circumscribed, solitary, firm, and painless nature of the mass.

The diagnostic approach followed in this case focussed on imaging and histopathological evaluation. Core needle biopsy or fine needle aspiration cytology is recommended by various studies prior to the removal of mass to exclude malignancy. Considering the benign outlook of the mass, direct surgical excision was performed in our case upon the patient's request after explaining the minor possibility of malignancy. The histopathological examination confirmed the diagnosis of vulval fibroadenoma, probably originating from mammary-like anogenital glands, as no ectopic mammary tissue was observed in the slides.

The recurrence rate of vulval fibroadenomas remains difficult to establish due to the limited number of cases reported in the literature. Li G et al. observed recurrence in only 3% of the cases [13]. Factors contributing to recurrence include tumor size exceeding 2 cm, hormonal exposure during pregnancy, lactation, or puberty, and iatrogenic factors such as ovarian stimulation or residual lesions. Ensuring complete resection with clear and safe margins is critical for reducing the risk of recurrence and allowing for adequate histological analysis of surrounding tissue.

## Conclusions

This case aims to create awareness about this rare condition and contribute to the cases reported previously. It also expands the clinical spectrum of vulval fibroadenomas, demonstrating the potential for pedunculated morphology and its occurrence without any typical hormonal associations. The findings reinforce the need for further research into the histogenesis of these lesions by ensuring the inclusion of peripheral tissues during the excision of vulval masses.
